# Bibliographical Notices

**Published:** 1856-07

**Authors:** 


### BIBLIOGRAPHICAL NOTICES.

---

*The Medical Profession in Ancient Times. An Anniversary Discourse delivered before the New York Academy of Medicine, November* 7, 1855. By JOHN WATSON, M. D., Surgeon to the New York Hospital. (*Published by order of the Academy.*) New York: Printed for the Academy by Baker & Godwin. 1856.

We are indebted to the politeness of the accomplished and learned author for a copy of the above Discourse, delivered last year before the New York Academy of Medicine. The work consists of thirteen chapters, in twelve of which the condition of Medicine is gradually traced from the earliest period in which it existed in organized communities down to the Latin and Greek Medical writers, subsequent to Galen; the thirteenth chapter containing an interesting account of the laws and customs of the Roman Empire in relation to our profession. "Though small," says the author, "the work is the result of no inconsiderable research; much of which might have been spared, had I been disposed to rely simply on the historians. But as my investigations were undertaken for my own gratification, I have, as far as leisure and opportunity would permit, drawn the facts and opinions here embodied, from the earliest authorities."

We need hardly observe that it is the duty of every physician to know somewhat of the past history of his science. To possess some information regarding the successive phases through which it has passed, some acquaintance with the history of its master-minds and their various doctrines, must be allowed by every one, we think, to be a proper and graceful, if not an essential component of the education of every member of the profession. How seldom, however, do we find such studies cultivated in the present day, even by physicians who have been liberally educated! This ungrateful and unwise neglect of the past is a marked feature of the present generation, which is too apt to regard whatever is antiquated as unprofitable and beneath the dignity of its notice—

> "And only wish
> As duteous sons, their fathers were more wise."

In speaking highly of such studies, however, we do not wish to be understood as upholding the view that the medical opinions of the ancients are proper guides to us either in the recognition or treatment of disease. The ardent spirit of investigation which characterises the present age has nowhere been more strongly shown than in the improvements it has imparted to our own science, and there is no reason to doubt that even the most learned and accomplished of the ancients, in his knowledge of medicine, was far beneath the humblest practitioner of the present day. The benefits of such studies lie in other directions; they constitute an excellent training and discipline of the intellect, and by their antagonism to that narrowness of mind which so frequently follows a complete devotion to any one special pursuit, their influence will be found to be both liberalizing and strengthening. They are the sources, also, of much happiness to those who possess a taste for them. "It is pleasant," as Dr. Watson remarks, "as well as profitable to turn, on fit occasions, from the bustle of active life, to the study of the past,—to the origin of our art, to the principles and necessities that called it into being, to the struggles of our ancestry. We are thereby better able to understand our position, to know how far we have advanced, to whom we owe our progress, the labor still before us, and the places we ourselves are likely to occupy in the estimation of those who are to follow us."

It cannot be denied that much of the respect which is paid us by the public is due to the presumption that we belong to a learned profession. The public, however, does not regard acquirements, great though they may be, in our own special department, as learning. As we have been educated to be physicians, it is naturally expected of us that we should understand the practice of our own science, just as it is expected of one who is brought up to a trade, that he should understand his trade. Something else is required to secure and sustain for us the reputation of being learned. It is gratifying, to find by such works as the one before us that there are still among us gentlemen, actively employed, too, in ministering to the infirmities of their fellow beings, who can fully uphold our reputation in this respect. We hail the appearance of such works as the 'Discourse,' therefore, with the greatest pleasure. That devotion to the *humanities*, as they are significantly called, is not incompatible, also, with the exercise of more urgent duties, is fully proven in the present instance, and we could add to it, were it necessary, the names of other gentlemen who are among the foremost practitioners of our profession.

As a specimen of the work we extract the following from the third chapter on the Asclepiadæ.

"As a rational study, so long as medicine was taught orally, or by tradition and example only, the acquirements of its votaries could not have been extensive. Their main study in the management of acute diseases, was in regulating the regimen. Epidemic diseases they looked upon as divine dispensations, with which they did not dare to interfere. A knowledge of the general rules of health, and the influence of diet, exercise, climate, and locality, attracted much of their attention. In the management of injuries and external diseases, they were but little inferior to their descendants of modern times. Their medical agents were the lancet, of which they made frequent use; certain active cathartics, emetics, and diuretics; cataplasms, unguents, escharotics; and mechanical instruments and appliances. Of anatomy and physiology their knowledge was limited; and as for chronic diseases, up to the time of Herodicus of Selymbria, who is said to have been one of the teachers of Hippocrates, they did not venture to interfere with them.

"This Herodicus had been a teacher of youth, and being always in delicate health, he had prolonged his life by systematic exercises and a regulated diet. The treatment which he had found useful in his own case, he recommended to others; and thus he turned the attention of medical men to a course of practice, and a group of diseases, which they had hitherto disregarded. His innovations were for a time unpopular; and even Plato undertakes to upbraid him for them, declaring 'that no attempt should be made to cure a thoroughly diseased system, and so to afford a long and miserable life to the man himself, as well as to his descendants. For Æsculapius,' he continues, 'did not think a man ought to be cured who could not live in the ordinary course, as in this case he would be of no service to himself or to the state.' He goes on to deplore the necessity of using the terms then recently invented for designating chronic diseases:—'Dropsies, and Catarrhs! Do not you think these abominable? Truly these are very strange names of diseases; such, I think, as existed not in the days of Æsculapius.' But though not in the habit of treating chronic internal ailments, the profession were at least supposed to be accquinted with them, so far as to be able to detect them, and pronounce correctly in regard to them, in the inspection of slaves. Even Plato would hold the physician responsible for his opinion in such cases, the object of the philosopher being to guard against dishonesty in the sale or transfer of slaves from one master to another.

"With respect, then, to the policy and ethics of the Asclepiadæ, we learn from the Oath and Law, as also from other passages in the Hippocratic code, that the student was formally bound to his master by indentures; that the son of a former master, choosing to enter the profession, received his education gratuitously; that others not thus circumstanced, were expected to pay for their instruction; that the sons of the Asclepiadæ did not necessarily follow their fathers' employment; that those who were employed in the temples, or in practice elsewhere, were therefore, simply a fraternity, in the modern acceptation of that word, and not, as some suppose, an exclusive caste derived from one family; that each practitioner was at liberty to follow his occupation where and when he chose, but for honorable purposes only; and that even at this early day, there were designing men who were 'physicians only in name,' and who gave themselves up to disreputable practices; against whom the regularly initiated had no redress, and no other advantage than that upon which we ourselves rely, a superior education, honesty of purpose, devotion to their duties, and the confidence of a discerning public."

Of Hippocrates, the writer says:

"Many speculations have been afforded, to account for the rapid advancement of medicine in the hands of this great father of the profession. According to Celsus, his principal credit was in removing the teaching of medicine from the school of philosophy, where it had always received some attention, and treating of it apart, as a distinct department of practical knowledge. Pliny, after Varro, supposes that he was the first to institute clinical instruction, '*hanc quæ Clinicæ vocatur,*' that he was led to this after the burning of the temple of Cos, and that the materials of his course were supplied mainly by the votive tablets which had been there accumulating. But his claim to our respect rests on higher ground.

The account of the museum at Alexandria and its occupants is very graphic; we regret that we cannot lay the whole description before our readers:

"But, among the public buildings of the rising capital, that which has the greatest claim upon our attention, and to which the city ultimately owed its fairest frame, was the Museum. This stood in the quarter of the Bruchium, fronting the harbor. Its chief apartment was a great hall, which served as a lecture-room and place of general concourse. Around the main building, on the outside, was a covered walk or portico; and connected with it, was an Exhedra, in which the philosophers sometimes sat in the open air."

"The men of learning in the several faculties of this institution, lived together in a sort of fraternity, eating at a common table, supported in whole or in part at the public expense. Some of them officiated as professors under a fixed salary; some of them as private tutors, deriving at least a portion of their income from their pupils; some of them were engaged in the public works, or in the service of the state; and some, as original investigators in the arts, in literature or philosophy, or in works of fancy, in the exact sciences, in natural history, or in medicine.

"Under Ptolemy Philadelphus, the Museum had already risen to the highest rank among the Greek schools. Its library already held two hundred thousand rolls of papyrus, equal to about ten thousand of our modern printed volumes. At the head of this library, under Ptolemy Soter, its founder, was Demetrius Phalereus, who had formerly been chief-magistrate of Athens. The system of instruction, as at first arranged, was divided among the four faculties of literature, mathematics, astronomy, and medicine; but other faculties, or special departments, must have been early adjoined to these.

"At the head of the mathematical department was Euclid. In that of poetry were Theocritus and Callimachus. The chair of philosophy was assigned to Hegesias of Cyrene, that of astronomy to Timocharis. The department of natural history was under Philostephanus, then engaged in a work on the history of fishes. Manetho, an aboriginal Egyptian, was occupied in preparing an elaborate history of his own country; and Timosthenes, the commander of the fleet, had in charge the subject of geography. In the medical faculty were Cleombrotus of Cos,

Herophilus, and Erasistratus. The first of these was in high repute as a practitioner; was sent to the relief of Antiochus when dangerously ill, and after curing that king, received on his return a present of a hundred talents, about fifteen thousand pounds sterling, as a reward from Philadelphus.

"Ptolemy Soter was himself an author, and the biographer of Alexander. As a man of enlightened understanding and cultivated taste, he took delight in the society of the Museum. He was in daily intercourse with the philosophers, listening to their discourses in the lecture-room, or entertaining them at his own table. At one of these literary dinners he is said to have asked Euclid for a shorter way to the higher mathematics than that by which the pupils were led in the lecture-room; when Euclid, as if to remind him of the royal roads of Persia which ran by the side of the public highways, but were kept clear and free for the king's use,—gave him the well-known reply, that there is no royal road to geometry.

"Among the rhetoricians of the museum was the sophist Diodotus Cronus, with whom Ptolemy was in the habit of jesting, and who among other paradoxes maintained the non-existence of motion,—arguing that motion was neither in the place from which bodies moved, nor in that to which bodies moved, and consequently had no existence. Cronus, however, by a fall, dislocated his shoulder; and when asked by Herophilus, who had been called to assist him, whether the fall had occurred at the place where the shoulder now was, or at that from which it had descended, was by no means contented with the application of his own argument, and begged the physician to begin at once by adjusting the dislocation.

"The seven ablest literary men at the Museum, were called the Pleiades; and they had in charge the business of adjudging prizes and rewards to the pupils. At one of their public sessions, a chair accidentally vacant among them, was, for the moment, assigned to the grammarian Aristophanes. When the reading of the exercises was ended, and most of those present were agreed upon the one deemed best of all the compositions, Aristophanes dissented from the general judgment, and pointed to the very volume in the library from which this performance had been copied. Ptolemy was struck with this test of the grammarian's acquirements, and soon afterwards promoted him to the post of librarian, then the most honorable office of the Museum. The Ptolmies reserved to themselves the right of appointment to office, and occasionally silenced the professors. Hegesias, in the midst of a discourse against the fear of death, was thus silenced, lest by his eloquence he might excite a passion for suicide among his hearers. But, while watching with solicitude over the business of oral instruction, they took no official notice of books. And Hegesias, no longer able to lecture, consoled himself by recording his opinions, and circulating them among his friends.

"At a time when books were expensive and readers few, the influence of private reading could hardly be felt upon the social institutions or political destiny of the nation; and hence it was disregarded. Not so with oral instruction. Among the Greeks this had always been the common mode of enlightening the people, of amusing them, and of molding their opinions. Most of the poetry, and much of the written history of the nation, were prepared for public recitation. Plato, aware of the influence of such exercises, would have had a censorship upon the poets, that they might not be permitted to recite their compositions in public before submitting them to the judges and guardians of the law, and obtaining their approbation. The busines of lecturing, therefore, was at Alexandria, as in the other cities, of more importance than that of composing for the private reader. The custom of appointing readers for familiarizing the people with Homer and other standard authors, had already been introduced here. And Hegesias, after the loss of his professorial chair, was occupied as the official reader of Herodotus."

We do not know whether the "Discourse" is for sale; if it be, we advise our readers to possess themselves of a copy of it. It is extremely interesting, very well written, and bears ample testimony throughout its pages to the erudition and industry of its author.

---

*Manual of Chemical Physiology. From the German of* Prof. C. G. Lehmann, M. D. *Translated with notes and additions by* J. Cheston Morris, M. D. *With an Introductory Essay on Vital Force.* By Samuel Jackson, M. D., Professor of Institutes of Medicine in the University of Pennsylvania. Illustrated with forty wood cuts. Philada.; Blanchard & Lea. 1856.

The above manual is a digest or abridgement of the author's large work on Physiological Chemistry, of which our readers will remember we gave a very full and elaborate notice in the February number. In the present work, Dr. Lehmann has endeavored "to place together, in as compressed a form as possible, the positive facts which can now be looked on as the certain possessions of physiological chemistry, and to bring to bear only those conclusions which carry upon them, according to our present physical views, the stamp of relative truth." In endeavoring to obtain the "compressed brevity" he had in view, the author was obliged to omit much of the matter, principally argumentative, however, of his larger work; and he leaves it to his readers to decide "whether the attempt to present the department of physiological chemistry in a short epitome, when the fixed marks are so few and the deficiencies so innumerable, has succeeded." A good abridgement is always valuable; its value is of course much greater when prepared by its author, than when done by any other person, as he can best judge how far the work can be reduced without losing its essential characteristics. The merit of the present work is enhanced also, by the fact that it contains the author's latest views, being essentially the same as those which are contained in his last unabridged work, the Sydenham Society translation and its American reprint being, as we have remarked in a former notice, a translation of a previous edition.

It gives us pleasure to state that the text of the author has been admirably rendered into English by the translator, Dr. Morris. The translation in fact is equal in every respect to the one made under the auspices of the Sydenham Society of the author's larger work, and gives evidence throughout of careful elaboration.

"From Dr. Lehmann's views of the forces operative in living organisms, (says Dr. Morris,) I must express my dissent. Dr. Jackson has been kind enough, at my request, to prepare an article on these views, stating the doctrine he has so ably advocated for many years.

"To adapt the work for the use of students of physiology, I have incorporated in the text additional matter (derived mainly from notes on Dr. Jackson's Lectures, Carpenter's Human Physiology, Todd and Bowman's Physiological Anatomy, Kölliker's Microscopic Anatomy, &c.,) of a more purely physiological nature. Short notes have also been added, in the shape of an Appendix, on kindred subjects not treated of by the author; and illustrations selected from various sources have been introduced, instead of referring, as the author has done, to the 'Atlas of Physiological Chemistry, by Otto Funke.' These alterations have so changed the character of the work as to render the title of 'Chemical Physiology' more applicable than that originally given to it of 'Handbook of Physiological Chemistry,' which has, however, been retained for Dr. Lehmann's portion of it."

The following are some of Dr. Jackson's remarks on Dr. Lehmann's doctrine of vital forces:

"The identity of the chemical actions of living beings and of inorganic bodies is now generally admitted. It is granted 'that the same chemical laws preside over the constitution and transformations of different compounds, whether organic or inorganic.' Admitting the accuracy of this proposition, it does not authorize the assertion, that 'all the differences observed between these two different classes of bodies are accidental, relative, and have nothing essential.' Will it be affirmed that, when an unfecundated egg is placed in the conditions for incubation, and the result is its putrefaction, while, in a fecundated egg, the albuminoid contents are transformed into blood, muscles, viscera, nerves, brain, heart, vessels and organs of sense, and it is endowed with special sensibilities, with consciousness and voluntary movements, that these two classes of phenomena are only accidental, relative and non-essential?

"It is certainly true, that chemical affinities and molecular actions are indispensable to produce the varied special proximate organic materials of the fluids, tissues, organs and the living organism of the chick. But, without the presence of the germ, a fact neglected by Dr. Lehmann, this extraordinary play of specific chemical affinities and specialities of chemical actions, and the development of some hundred organic forms, included in the living being, from the formless organic matter, could not have occurred. Certainly, here are displayed, in the two classes of bodies—inorganic and organic—differences that are not accidental, or relative, and that are essential.

"The same general facts are observed in the germination of seeds. If the germ cell at the hilum of a seed be artificially ruptured, and it be placed in the same circumstances of germination with a perfect seed, the first will rot, while in the other are formed dextrin, grape-sugar, cellulose and albuminoid compounds; and these organic matters are developed into the tissues and organs of a perfect plant.

"In these, which are but single series of organic phenomena, the most absolutely marked and undeniable differences are to be observed between the two classes; it is impossible to regard them as 'accidental, relative and non-essential.'

"These phenomena have occurred in living beings with absolute constancy, in all generations of animals and vegetables, and are so completely under the control of positive law, that any one, or all of them, can be predicated and foretold with as much certainty as any chemical or physical action.

"Another argument urged as conclusive is the following: 'We should not lay down a new force, a general specific cause, until we have eliminated all other possibly operative forces from the group of phenomena in question. A proof of the existence of a purely vital force is hence only to be obtained by an exclusion of every physical force.' A full assent is given to the conditions of the argument; and it is affirmed most positively that the only phenomena—the exclusive attributes of a vital or organic force—the creation from a liquid formless plasm of typical organic forms, and organic instruments to execute physical, chemical, mechanical and dynamical actions, in the living organism, which are operative only during life, cannot be explained by any one, or all of the physical forces combined. They are absolutely excluded. Let the subject be expressed in special terms, and it is affirmed that neither heat, nor light, nor electricity, nor magnetism, nor gravity, can construct an eye, can form a retina, heterogeneous in its organic materials, complicated in the distinct organized anatomical elements of its structure, and endowed with a special sensibility—light and colors—manifested normally only when excited by a special external agent, the luminiferous ether. Nor can they generate the black pigment cells of the choroid coat, for the express purpose of suppressing the luminous rays, which have accomplished their impression, without which there can be no distinct vision; nor cause the formation anterior to the retina of the vitreous humor and the crystalline lens, admirably adapted in form, shape and density, to refract the rays of light into a focus, the distance from the retina calculated so truly, as to subtend on it a visual focus, in which is a perfect representation of external bodies. The formation of this organic optical apparatus, constructed in the fœtus included in the ovum or uterus, excluded from the direct operations of the physical forces except heat, it is affirmed, is absolutely inexplicable by the physical forces, or by any known physical process or actions.

"The insuperable difficulties presented by these facts, it is sought to evade by assuming that, as the causes of physical actions are unknown, and that new physical phenomena may be discovered, a resort to a new force is useless and unscientific. But it will not be pretended that the causes of physical actions will ever be better known than they are at present; and, whatever new physical actions may be discovered, they cannot differ in their nature from those of the present known physical actions. They will be unable to furnish any new light to solve the nature and origin of phenomena that reach far beyond the range of the physical forces; to which physical forces and actions are accessories, but cannot hold the relation of productive causes.

"From the admission by Dr. Lehmann, in his argument 'that a new force may be assumed in science, whenever groups of phenomena are inexplicable by any known forces,' the assumption of an organic or vital force, as distinct from physical forces, and as presiding over the developments of the organic forms of living beings, is an unauthorized and legitimate deduction.

"A third argument, and the last that will be noticed as having a bearing on the question is, 'that the idea of a vital force is illogical; for a force is merely the abbreviated expression of a law from which the casual connection of certain phenomena may be deduced; and that a vital force corresponds to no law.'

"This statement proves that Dr. Lehmann has not investigated the physiological facts of embryology or organic development, or he could not have so broadly asserted that the vital or organic force corresponds to no law, and is not a necessary cause of multitudinous consequent phenomena.

"So far is this statement from being correct, it may confidently be asserted that the evidences of law, of casual connection and dependence are as strong, as palpable, in the phenomena, the direct results of organic or vital force—those of organization—as are to be found in any of the physical forces. A few facts will prove this position. Prevent the spermatozoon from reaching the egg, no monadiform germ cell, the primary form of all animals, is produced. Let this germ cell be artificially broken or injured, and no blastoderm will be formed; injure the blastoderm, and either no embryon and chick will be developed, or this last will be imperfect.

"Here are law and causal dependence and connection. Professor Owen, in his *Hunterian Lectures*, (ed. 1855,) has demonstrated that 'every animal, in the course of its development, represents some of the permanent forms of animals inferior to itself, but it does not successively repeat them all, nor acquire the organization of any of the inferior forms which it transitorily typifies. One organic form, the microscopic infusorial monad, is either permanently or transitorily represented throughout the animal kingdom. Other forms are represented less exclusively in the development of the animal kingdom, and may be regarded as secondary forms. These are the polype, the worm, the tunicary and the lamprey. They are secondary in relation to the kingdom at large, but are primary in respect to the primary divisions or provinces."

"In dissenting from the author's views of organic or life force, there has been no purpose of disrespect, or intention to undervalue his knowledge and the authority of his opinions on the special branch he has cultivated. It has been rendered necessary by the manner in which the subject has been treated. Almost exclusively physiological, it has been handled as a chemical question. All that has been urged by the author as to the extent, variety and importance of the chemical actions and influences of chemical laws in the living organism is perfectly just; but to force chemical affinity and its laws from their appointed ordinances in nature, is not correctly scientific, and leads to serious errors and misconceptions. Yet such a perversion, it strikes us, takes place when physics and chemistry are summoned to explain the origin and the permanent constitution of the typical forms of organization, differing so widely from the molecular processes and actions, exclusively the results of their special activity and energies in the plan of creation.

In adopting the handbook of Dr. Lehmann as a manual of organic chemistry for the use of the students of the University, and in recommending his original work of *Physiological Chemistry* for their more mature studies, the high value of his researches and the great weight of his authority, in that important department of medical science, are fully recognized."

We have quoted Dr. Jackson's views very fully on this question; they are extremely interesting, and we have no doubt they will bring conviction to the minds of most of his readers. We are not entirely satisfied with them, however, as it appears to us that he has trusted too much to the present work, which, as previously stated, is but an abridgment of the larger one; we submit the following, therefore, as a synopsis of what we conceive to be Dr. Lehmann's views:

Webster defines vitality as being, "first, the *principle* of life, and, second, as the *act* of living;" thus confounding, or rather combining in the same word the hidden cause that produces the action, with the manifestation of the act itself. This complication of ideas arising from the two-fold significance of the term, has extended even to those whose study is the laws of life, and has produced endless and profitless disputes about *theories* and *systems*, when the point at issue in reality has only been the significance of a *word*. Dr. Lehmann, whilst admitting the necessity of a *great first cause* and of a *creator*, and rejecting as absurd the possibility of a spontaneous generation, or the growth of an organic existence from any other source than its germ, believes that we should not waste time in seeking for the *source* of life, but study the phenomena of *existing* life through the same medium that has already supported us thus far in the investigation of the works of God, viz., the laws of physics and of chemistry.

It is true that almost at the commencement of our investigations into the mysteries of life, we find metamorphoses and changes taking place which our stock of knowledge is utterly insufficient to explain, and as we advance deeper into the subject we soon lose the little light that has thus far guided us, and find ourselves in total ignorance of all around us. This condition of things operates differently on differently constituted minds, and whilst it causes some men to believe that the search is a right and just one, and that the path thus far followed must lead, if diligently pursued, to the utmost limit of knowledge allowed to mortal man, causes others to think the hope is futile of ever explaining the difficulties that oppose us, for that here the fixed and immutable laws of chemistry, etc., no longer reign, being deposed by the capricious rule of the *vital forces*, whose attributes are irregularity, uncertainty and mystery.

Among the numerous errors into which the mind of man is prone to fall, none have more frequently obstructed the progress of science than the practice of condensing into a single word or phrase the description of a law of nature or the manifestation of its action, and by thus investing as with a garment what is often merely a crude imperfect hypothesis, obtain for it a credence and position that is utterly undeserved in fact. The evil does not end even here, for the temptation to employ a *word* instead of an *idea* causes many men of even high intellect to rest satisfied with its use without further inquiry, and thus disseminate error or, at least, uncertainty, almost unconsciously. Often, it is true, such a term may rightfully include our whole knowledge of the subject, and when expressed may give utterance to all that we could say. Thus, the laws of gravity or the attraction of gravitation are terms that admit of little amplification, since the *cause* of the attraction of matter by matter is an enigma that would be idle to attempt to solve. But the theory of catalysis, for instance, rests on a different foundation. It is at best but a term by which is understood a group of phenomena whose similarity consists in being alike inexplicable by known laws, from which group, however, the advances of science withdraws yearly its most prominent members by reducing them to natural laws. It is in this latter category that we should place the doctrine of the vital forces, when it is used as significant, not of the fact of vitality or of the living spirit within the animal form, but of an incomprehensible group of actions that do not admit of being indicated distinctly, that have no connection with each other, nor apparent means of effecting the desired end, that in fact elude our observation, both in their modes of action and in the effect produced; in other words, force us to acknowledge that we can neither explain nor understand them, which is precisely the state of ignorance we are in by the assumption of this doctrine.

It is needless to say that the physiological chemist does not and ought not to confound the chemistry and physics of the organic body with the unknown principle of vitality that is contained within it. The sporule of the humblest cryptogamic plant contains an immaterial essence that is as much, indeed, even more, beyond the reach of our gross senses than that contained within the ovum of a mammal; for if we could explain *why* the globule of the yeast plant grows by gemmation when placed in its proper element, we would then have a position that would enable us eventually to trace the vital principle through the ascending series of creation even to man himself. We would be master of the secret of vitality! But the physiologist *can* hope, some distant day, to follow step by step the operations of *natural laws* from the first endosmotic current through the vesicles of the germinating seed, to the development of the aged and giant oak—from the first appearance of the ovum, to the growth of the perfect man. The *cause* of the vitality is the same—one and incomprehensible —GOD!

*The Causes and Curative Treatment of Sterility, with a preliminary statement of the Physiology of Generation. With colored Lithographs, and numerous wood-cut illustrations.* By AUGUSTUS K. GARDNER, A. M. M.D., Permanent Member of the National Medical Association ; Fellow of the New York Academy of Medicine ; Member of the Massachusetts Medical Society, &c., &c. Author of Monographs on Ergot, Uterine Hæmorrhage, Rupture of the Perinæum, &c. New York : De Witt and Davenport, 160 and 162 Nassau Street.

We notice this book merely because we think it a duty we owe to the profession to discourage the publication of valueless works, and likewise to record our decided opinion that medical aspirants to literary fame, whatever may be the amount of their experience in the treatment of disease, should, if they must write, take into consideration the vital necessity of studying the meaning of the phrases and words they are about to inflict upon the public, and have at least a superficial knowledge of the grammatical construction of their own language.

Had Dr. Gardner given us anything new, had he said any thing which has not been better said, many times before, we might have excused his violations of the rules of syntax, and his occasional chaotic obscurity; but unfortunately this confusion of words is unredeemed by a single suggestive or novel thought.

We should be sorry to be thought unfair or uncandid in our strictures; in truth we should have said but little of these comparatively minor faults, were it not for the coarseness with which the subject is frequently handled. Description is necessary, we know, but such a topic every gentleman must feel cannot be approached too delicately. To make such books endurable, the style should be pure and at the same time severely scientific; when thus filtered through a refined and educated intellect, their themes become ennobled by purity and benevolence ; divested of these elevating attributes, nothing remains but a grossness which offends and cannot instruct, which degrades a high calling in the minds of the educated and thinking portion of the community, and demoralizes by placing the fairest and gentlest of God's works before us as a mere machine for manipulation.

In order to prove that our remarks have not done injustice to Dr. Gardner, we must trouble our readers with a few quotations ; we promise to be as brief as possible.

In his preface we find the following : "Occasionally these conditions arise from a defect in the provisions of nature, almost always, however, are the consequences of disease."

The little pronoun *they* before the are, would have rendered this sentence grammatical.

The next sentence informs us that "Sometimes the difficulties have been suffered to continue unchecked, till organic changes are effected which can never be *palliated.*" The symptoms of organic changes may be palliated, but we think the word applied to organic change itself inadmissible.

Again, Dr. Gardner says, "that organic changes may be alleviated." We should prefer some other word here; *alleviation* being more applicable to symptoms or effects.

At page 11, of the introduction, we find this sentence : "All men are curious by nature. They love to look into the interiors of even man's feeble constructions."

At page 67, speaking of the causes of sterility, "Such for instance, are numerous forms of tumors, and of local or other manifestations of constitutional syphilis, which according to its location may or not form a barrier to fecundation.

At page 68, the Dr. observes that obliquity of the womb may militate *with* the healthy action of the rectum and bladder.

On page 94, he speaks of extra delicate ladies :

On page 111, he says, "According to my own views, the reasons given for the want of fertility in the previous remarks embrace all the causes that exist, but there are a train of causes alleged by other writers."

Page 109, "there is usually certain physical peculiarities," &c.

At page, 115, Dr. Gardner, treats his readers to the history of a precocious child exhibited at Barnum's baby show ; this child menstruated, or as our author expresses it had a bloody flow, at the age of ten months, which has continued periodically ever since. At four years of age "this flow," says Dr. G. "had been retarded for some two weeks longer than usual, and the mother seriously supposed her to be pregnant, as she had discovered a man (for which sex she evinced great fondness) in an improper situation respecting her, and she had him arrested, and he lay in prison several days on this charge. The flow however soon reappeared." Now such cases of precocity have been described before, and we think that our author would have evinced more taste by simply stating that such curiosities have existed, than by repeating this story, which is disagreeable and offensive in its character from Barnum down to the woman baby.

We should advise Dr. G., in his second edition, to expunge the very coarse sentence at the end of the second paragraph on page 115. The statement was not necessary, and its omission would greatly improve the general texture of the work. The remarks on page 132 are equally offensive. The passage on page 18, where the phrase "regular monthly bloody flow" is used, might we think without much trouble be rendered differently.

We quote the following passage merely as a curiosity in construction.

"Ante and retroversions are among the most troublesome forms of uterine disease, *and too,* are far more common than has been supposed."

We cannot afford to waste further time on this catologue, which might be extended almost *ad infinitum.* But we are satisfied that these few extracts will sufficiently sustain us in the opinion we have given, which is all that we require.

To conclude, the author is evidently a man of good perceptive powers, and considerable practical capability, and has studied carefully the best physiological writers on conception, generation, &c. He is, we have no doubt, a good practitioner; that is, he introduces catheters and bougies and forceps with great ease to himself and satisfaction to his patients. This is his vocation; we cordially wish him success in it, but as cordially we hope that he will write no more such books; his bad English we might have passed lightly over, but there is a want of delicacy in his phraseology which peculiarly unfits him for writing pleasantly or even readably on the subject he has chosen. When we first opened this volume the frontispiece astonished us, and was prophetic of all the coarseness which succeeds. As a sincere well wisher of Dr. Gardner, we would honestly advise him in his next edition to dispense with that curious and suggestive specimen of art.

*A Manual of the Practice of Medicine.* By GEORGE HILARO BARLOW, M. A. and M. D., Cantab. &c. *With additions, by D. Francis Condie, M. D., &c.* Philadelphia: Blanchard & Lea. 1856.

In accordance with the plan he has laid down for himself or the necessity by which he is bound, every author has the undoubted right to make his work either a full representation or a condensed outline of his subject; he may exercise his own pleasure, also, regarding the omission of certain topics, without his readers having any just right to complain; it is his duty, however, to give such a title to his work when finished as will afford all who wish to obtain it, a correct opinion of the nature of its contents. This Dr. Barlow has not done. He styles his work a Manual of the Practice of Medicine, and yet omits even the names of numerous diseases which the practitioner may be at any time called upon to encounter. Among these, we notice diseases of the skin, of the spleen, of the pancreas, gangrene of the lungs, emphysema, and a host of others equally important. This want, the American editor has in some degree supplied it is true, by the addition of articles on Cholera Infantum, Cerebro-spinal Meningitis and Yellow Fever; yet even with these aids, as a Manual of the Practice of Medicine it must be allowed to be exceedingly incomplete.

It is evident, also, that the author has devoted much more time and labor to some subjects than to others. The articles, for instance, on Phthisis and Diseases of the Heart and Liver, are extremely well written and full for such a work, and may be consulted with profit by practitioners as well as students. Bright's disease is also fully discussed. In the treatment of other affections, hooping-cough, epidemic cholera, &c., he is extremely meagre and unsatisfactory. The following are his conclusions regarding the identity of typhus and typhoid fever—both of which diseases he treats of under one head: "The conclusion," he says, "which it appears we may most legitimately draw from our present information upon this subject is, that in the fevers in which the mulberry-colored and livid spots are present, there is a greater tendency than in others to assume the low, sinking form, and perhaps a greater liability to head affections, but that nevertheless there may and frequently does occur severe bowel irritation, with inflammation and ulceration of the lower portion of the ileum. When there is the rose colored rash, on the other hand, there is almost always great bowel irritation, and not such early depression from the effects of the poison ; but the frequency with which one form of the disease has been found to occur side by side with the other in many epidemics, though it may not have done so in all, and the almost imperceptible difference by which they appear to be distinguished in some instances, seems at present to preclude the belief that they are specifically different." From this opinion we dissent *in toto.*

In spite of the short-comings we have pointed out, we feel ourselves justified in recommending Dr. Barlow's work as the production of an experienced and judicious practitioner, one whose knowledge of disease and its proper treatment has evidently been obtained less from books than from practical experience at the bedside. Few physicians, we imagine, will rise from the perusal of this manual, without receiving some instruction from it. The American Editor's additions add greatly to the value of the work.

